# Methyltransferase-Like 3 Rescues the Amyloid-beta protein-Induced Reduction of Activity-Regulated Cytoskeleton Associated Protein Expression *via* YTHDF1-Dependent N6-Methyladenosine Modification

**DOI:** 10.3389/fnagi.2022.890134

**Published:** 2022-04-25

**Authors:** Chenhaoyi Xu, Huanghuang Huang, Min Zhang, Pei Zhang, Zezhi Li, Xueyuan Liu, Min Fang

**Affiliations:** ^1^Department of Neurology, Shanghai Tenth People’s Hospital, Tongji University School of Medicine, Shanghai, China; ^2^The Affiliated Brain Hospital of Guangzhou Medical University, Guangzhou, China; ^3^Guangdong Engineering Technology Research Center for Translational Medicine of Mental Disorders, Guangzhou, China

**Keywords:** N6-methyladenosine (m^6^A), methyltransferase-like 3 (METTL3), YTH domain family, member 1 (YTHDF1), activity-regulated cytoskeleton-associated protein (ARC), Alzheimer’s Disease (AD)

## Abstract

Activity-regulated cytoskeleton-associated protein (ARC) is activated by the induction of long-term potentiation and plays an important role in the synaptic plasticity of memory consolidation. Previous studies have shown that abnormal expression of ARC in the brains of patients with Alzheimer’s Disease (AD) leads to the disturbance of synaptic plasticity. ARC expression is mainly regulated by transcriptional and post-translational modification. However, it is unclear whether N6-methyladenosine (m^6^A) engages in the epigenetic modification of *ARC*. The AlzData database was used to analyze the brain of AD patients, and Aβ-induced cell models were used. We revealed that ARC expression was reduced in AD patients and Aβ-induced cell models. There were five m^6^A modification sites of *ARC* mRNA that were predicted by the SRAMP database, and *ARC* mRNA was confirmed as the target gene of methyltransferase-like 3 (METTL3) by MeRIP. Amyloid-beta protein (Aβ) repressed the m^6^A modification. Knockdown of METTL3 decreased *ARC* mRNA m^6^A modification and reduced ARC protein expression, while overexpression of METTL3 rescued ARC expression after Aβ treatment. Knockdown of YTH domain family, member 1 (YTHDF1) decreased ARC protein expression, while the overexpression of YTHDF1 could not rescue the loss of ARC protein expression after 3-deazaadenosine treatment or knockdown of METTL3. Our findings identify that METTL3 rescues the Aβ-induced reduction of ARC expression *via* YTHDF1-Dependent m^6^A modification, which suggests an important mechanism of epigenetic alteration in AD.

## Introduction

Alzheimer’s Disease (AD) is mainly characterized by a progressive loss of memory and cognitive functions. Accumulating evidence has shown that the deposition of amyloid-beta protein (Aβ) may interfere with neuronal communication *via* the synaptic structure and lead to short-term or long-term alterations in the neurons, resulting in neuronal injury and apoptosis (Styr and Slutsky, 2018). It has been proven that epigenetic regulation plays a critical role in maintaining stable synaptic plasticity, which is required for memory (Day and Sweatt, 2011; Kim and Kaang, 2017). Furthermore, the formation of long-term memory is accompanied by the expression of immediate early genes (IEGs). Studies have reported that the deficiency of IEGs represents common epigenetic changes observed in AD models, which indicates that IEGs contribute to the pathogenesis of AD (Anderson et al., 1994; Dickey et al., 2004).

Activity-regulated cytoskeleton-associated protein (ARC), also known as activity-regulated gene 3.1 protein homolog (ARG3.1), is a member of IEGs. ARC can be activated by the induction of long-term potentiation and plays an important role in the synaptic plasticity of memory consolidation (Kedrov et al., 2019). It has been reported that the newly synthesized *ARC* mRNA can be rapidly transported to dendrites and then later translated and enriched in the post-synaptic density (Steward et al., 1998; Newpher et al., 2018). ARC protein mainly promotes the endocytosis of the α-amino-3-hydroxy-5-methyl-4-isoxazolepropionicacid (AMPA) receptor on the post-synaptic membrane by interacting with cytoplasmic proteins, thus inducing long-term depression (Diering and Huganir, 2018). Confusingly, the relationship between ARC and AD is the opposite. For example, ARC protein expression decreases in the dentate gyrus and CA1 region of AD mice, and the neurons with amyloid plaque-associated dystrophic neurites fail to express *ARC* mRNA (Chowdhury et al., 2006; Rial Verde et al., 2006; Shepherd et al., 2006). Conversely, ARC increases in the medial prefrontal cortex of AD brains, contributing to the generation of Aβ (Wu et al., 2011). This evidence indicates that either the absence or overexpression of ARC may cause abnormal excitability of the neural network. Therefore, to elucidate the precise mechanisms underlying the regulation of ARC, it is crucial to elucidate the relationship between ARC and AD.

Besides being regulated by transcription and translation, ARC expression is also regulated by complex, flexible transcriptional or post-translational modification. Studies have reported that the SUMOylation, ubiquitination, and phosphorylation of ARC proteins could enable ARC to quickly respond to synaptic signals and maintain synaptic plasticity (Nikolaienko et al., 2017; Walczyk-Mooradally et al., 2021). In particular, epigenetic modification, such as DNA methylation, histone methylation, and histone acetylation, engages in the regulation of ARC (Penner et al., 2011; Singh and Thakur, 2018; Myrum et al., 2020). In recent years, RNA N6-methyladenosine (m^6^A) modification has been shown as a new epitranscriptomic modification, due to its similarities to epigenetic DNA and histone modification (Zhang et al., 2020). As the most prevalent modifier of eukaryotic mRNA, m^6^A modification is a dynamic course that includes methylation, demethylation, and recognition of m^6^A mRNAs (Zhao et al., 2017). The process of m^6^A modification requires three categories of proteins: methyltransferases (“writers”), demethylases (“erasers”), and proteins that can bind to m^6^A (“readers”) (Yang et al., 2018). The “m^6^A writers” complex [methyltransferase-like 3-methyltransferase-like 14-WT1 associated protein (METTL3–METTL14–WTAP)] transfers the methyl group from *S*-adenosyl methionine to mRNA, thus forming m^6^A modification (Liu et al., 2014). The “m^6^A readers” comprise of the YTH domain family members (YTHDFs) and include YTHDF1, YTHDF2, and YTHDF3. They recognize the m^6^A sites and accelerate the metabolism of m^6^A-modified mRNA through alternative splicing, translation, and decay (Zaccara and Jaffrey, 2020). Several studies have revealed m^6^A’s involvement in regulating the formation and consolidation of memory, and abnormality of m^6^A is involved in AD, but the specific functions of m^6^A modification in the process of memory remain to be illustrated (Zhang et al., 2018; Huang et al., 2020; Shafik et al., 2021; Zhao et al., 2021). Furthermore, whether m^6^A modification can regulate *ARC* expression during the process of AD hasn’t been investigated.

Thus, the purpose of this study was to investigate the potential mechanism of m^6^A modification in regulating ARC expression in Aβ-induced models. The role of epigenetic alteration on ARC regulation of synaptic plasticity was explored by investigating the METTL3/YTHDF1 dysregulation in AD.

## Materials and Methods

### Analysis of Activity-Regulated Cytoskeleton Associated Protein Expression in Alzheimer’s Disease Patients

The expression of ARC in AD patients was analyzed by the AlzData database.^1^ The AlzData database is a public, one-step database that integrates the current high-throughput omics and genomics data of AD patients (Xu et al., 2018). The ARC expression results of individual studies or cross-platform normalized data were analyzed in different brain regions. The results were all adjusted for age and sex in the AlzData database.

### Primary Neuronal Isolation and Culture

The animals were purchased from Shanghai Laboratory Animal Center. Primary hippocampal neurons were collected from embryos of Sprague-Dawley rat as described previously (Lu et al., 2016). Then, 6 × 10^5^ cells were plated in 6-well plates coated by poly-L-lysine. The primary rat hippocampal neurons were cultured in Neurobasal Medium (Gibco, MA, United States, #21103-049) containing B-27 Serum-Free Supplement (Gibco, MA, United States, #17504-044) and GlutaMAX Supplement (Gibco, MA, United States, #17504-044) at 37°C with a mixed gas of 21% O_2_, 5% CO_2_ and N_2_. The study was approved by the local ethics committee of Tenth People’s Hospital, Tongji University School of Medicine, Shanghai (No. SHDSYY-2020-0971).

### Cell Culture and Transfection

Human neuroblastoma and primary rat hippocampal neurons were cultured at 37°C with mixed gas (21% O_2_, 5% CO_2_ and N_2)_. The SH-SY5Y cells were cultured in Dulbecco’s Modified Eagle’s Medium containing 10% Fetal Bovine Serum and 1% penicillin-streptomycin. For Aβ treatment, human Aβ_1–42_ (MCE, Shanghai, China, #HY-P1363) was dissolved, and the solution was incubated for 3 days at 37°C to form oligomeric Aβ, and then cells were treated with 10 μM Aβ for 48 h. The concentration of Aβ used in the study referred to our previous studies (Fang et al., 2019; Xu et al., 2021). The knockdown or overexpression of target genes was achieved by using Lipofectamine 3000 Reagent (Thermo Fisher, MA, United States, #L3000-015). For transfection, METTL3 siRNA and its negative control siRNA were synthesized by RiboBio (Guangzhou, China), and METTL3 cDNA, YTHDF1 siRNA, YTHDF1 cDNA, and their corresponding controls were synthesized by Gene Chem (Shanghai, China). The target sequence of METTL3 siRNA was CAGCTTCAGCAGTTCCTGAAT, and YTHDF1 siRNA was AACCTCCATCTTCGACGACTT.

### Cell Viability Assay

The viability of the SH-SY5Y cells was measured by Cell Counting Kit-8 (CCK-8; Beyotime, Shanghai, China, #C0041). 10 μl CCK-8 solution was added into the cells and incubated at 37°C. The cell viability was assayed by testing the absorbance at 450 nm.

### RNA Isolation and Real-Time Quantitative Polymerase Chain Reaction

Total RNA was extracted with Trizol reagent and transcribed reversely into cDNA using the PrimeScript RT Reagent Kit (Takara Bio, Shiga, Japan, #RR037A). Real-time PCR was performed using the TB Green Premix Ex Taq II Kit (Takara Bio, Shiga, Japan, #RR820A). The primers were synthesized by Tsingke Biological Technology (Beijing, China). The sequences of primers are shown in Supplementary Table 1. The mRNA expressions of target genes were normalized to that of β-actin as an internal reference.

### RNA Stability Assay

Cells were plated onto six-well plates and transfected with siRNA described above. After transfection for 48 h, cells were incubated with actinomycin D (MCE, Shanghai, China, #HY-17559) at 5 μg/ml at 0, 3, and 6 h. Total RNA was isolated for RT-qPCR.

### Western Blotting

Cells were treated with RIPA Lysis Buffer (Epizyme, Shanghai, China, #PC101) with 1 mM PMSF (Beyotime, Shanghai, China, #ST506-2). The lysate was then centrifuged, and the supernatants were collected. The protein concentration was quantified by Bicinchoninic Acid Assay (Beyotime, Shanghai, China, #P0011). Subsequently, 15 μg of protein were loaded and separated on 8% SDS gels and then transferred onto PVDF membranes. The membranes were blocked for 1 h, incubated with appropriate primary antibodies at 4°C overnight, and then incubated with corresponding secondary antibodies. The optical density (OD) of each band was quantified using ImageJ (NIH, MD, United States). Antibodies used in experiments were as follows: METTL3 (Abcam, MA, Unitied States, RRID: AB_2721254), YTHDF1 (Abcam, MA, Unitied States, RRID: AB_2892231), ARC (Proteintech, IL, United States, RRID: AB_2151832), GAPDH (Proteintech, IL, United States, RRID: AB2263076), Goat Anti-Rabbit IgG (Jackson ImmunoResearch, PA, United States, RRID: AB_2313567), and Goat Anti-Mouse IgG (Jackson ImmunoResearch, PA, United States, RRID: AB_2338447).

### Immunofluorescence Staining

The expression of ARC protein was examined by immunofluorescence staining in primary rat hippocampal neurons. The neurons were collected, fixed, blocked, and then incubated with ARC antibodies (Proteintech, IL, United States, RRID: AB_2151832) overnight. The cells were treated with secondary antibodies and DAPI on the following day. Finally, cells were observed and photographed with Confocal microscopy (Carl Zeiss, Oberkochen, Germany) at a magnification of 40×.

### Quantitative Analysis of N6-Methyladenosine Level

The m^6^A level of total RNA was quantified with the Colorimetric EpiQuik m^6^A RNA Methylation Quantification Kit (Epigentek, NY, United States, #P-9005) following the manufacturer’s instructions. In brief, 200 ng RNA was bound to each well using the RNA high binding solution. m^6^A is captured and detected by corresponding antibodies. Then, the OD of each sample was detected at 450 nm using a microplate spectrophotometer (Bio-Tek, VT, United States). The percentage of m^6^A in total RNA was calculated as follows: m^6^A% = (Sample OD – Negative Control OD) ÷ 200 ÷ (Positive Control OD – Negative Control OD) × 100%.

### Prediction of m^6^A Methylation Site

The m^6^A methylation site was predicted by the Sequence-based RNA Adenosine Methylation Sites Predictor (SRAMP).^2^ SRAMP is a public prediction tool based on a random forest machine learning framework (Zhou et al., 2016). The predictor takes the transcript sequence as the input and reports the possible m^6^A sites and confidence of each possible m^6^A site.

### N6-Methyladenosine-RNA Immunoprecipitation–Quantitative Polymerase Chain Reaction

The MeRIP was performed with the EpiQuik CUT&RUN m^6^A RNA Enrichment (MeRIP) Kit (Epigentek, NY, United States, #P-9018-24) following the manufacturer’s instructions. Briefly, RNA sequences on both ends of the target m^6^A-containing regions were cleaved, and the target m^6^A-containing fragments were pulled down using a beads-bound m^6^A capture antibody. Then, the enriched RNA was released, purified, and eluted for RT-qPCR.

### Statistical Analysis

No blinding or randomization was performed, and there was no test for outliers. All data was expressed as mean ± standard error of the mean (SEM). All experiments were independently repeated at least three times. Statistical analysis was done with SPSS version 22.0 (IBM, NY, United States). Two-tailed unpaired student’s *t*-test was conducted to compare data in two groups. Comparisons among three or more groups were done with analysis of variance, and the Bonferroni *post hoc* tests were conducted for correction. The expression of ARC in Alzdata was analyzed by Fisher’s exact test and the Benjamini-Hochberg’s method, according to its description (Xu et al., 2018). A value of *p* < 0.05 was considered statistically significant and *p* values coded as follows: *p < 0.05, **p < 0.01, ***p < 0.001.

## Results

### Deficiency of Activity-Regulated Cytoskeleton Associated Protein in Alzheimer’s Disease Patients and Amyloid-Beta Protein-Induced Cell Models

**FIGURE 1:**
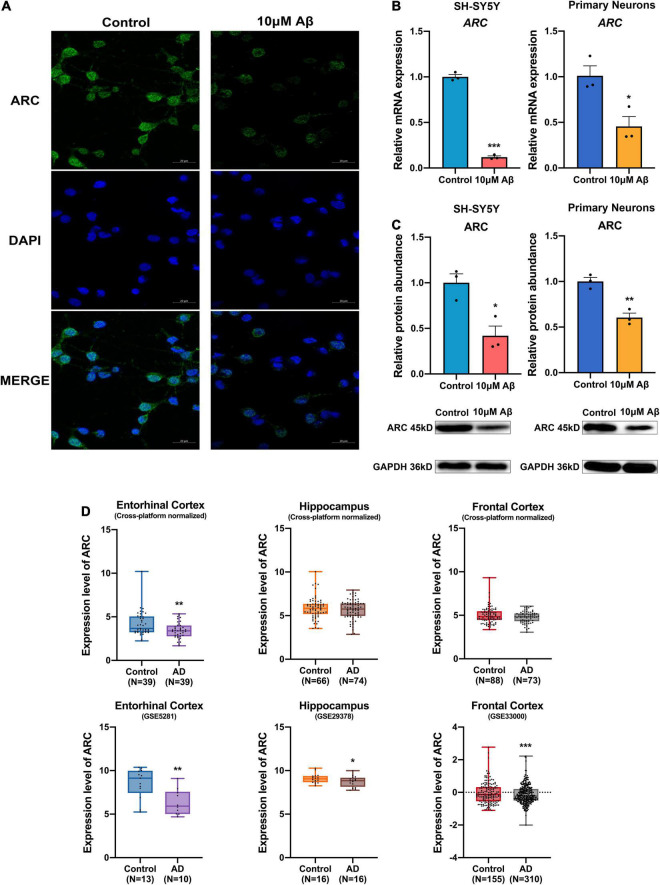
Decrease in activity-regulated cytoskeleton associated protein (ARC) expression in Alzheimer’s Disease (AD) patients and AD models. **(A)** Immunofluorescence showed lost ARC expression after 10 μM Aβ treatment. **(B)** qRT-PCR showed decreased mRNA levels of *ARC* after 10 μM Aβ treatment (*n* = 3). **(C)** Western blotting analysis showed declined protein levels of ARC after 10 μM Aβ treatment (*n* = 3). **(D)** The ARC expression results of individual studies or cross-platform normalized data in AD patients compared with those of healthy subjects in the AlzData database. Data **(B,C)** were expressed as mean ± SEM, and **(D)** boxes showed interquartile distance, lines indicated median, and whiskers indicated minimum to maximum. **(B,C)** Unpaired *t*-test, and **(D)** Fisher’s exact test and the Benjamini-Hochberg’s method were performed. *p < 0.05, **p < 0.01, ***p < 0.001.

Immunofluorescence staining of rat hippocampal neurons showed decreased ARC protein expression after treatment with Aβ (Figure 1A). Consistently, mRNA and protein expression of ARC also decreased in both SH-SY5Y cells and rat hippocampal neurons after Aβ treatment (Figures 1B,C). Moreover, ARC expression in AD brain was analyzed by the AlzData database (see text footnote 1). The cross-platform normalized data showed ARC simply decreased in the entorhinal cortex in AD brain, and some individual studies showed ARC decreased in the entorhinal cortex (GSE5281), the hippocampus (GSE29378), and the frontal cortex (GSE33000) in AD brain (Figure 1D and Supplementary Table 2).

### Down-Regulation of N6-Methyladenosine Modification Mediated by Methyltransferase-Like 3 in Amyloid-Beta Protein-Induced Cell Models

**FIGURE 2:**
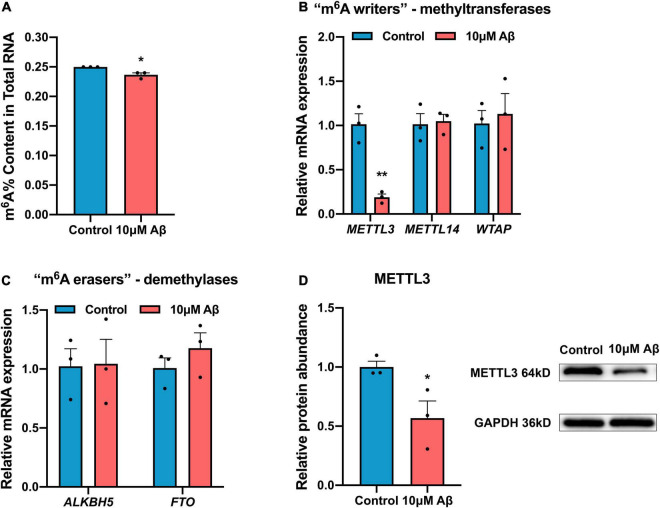
Down-regulation of m^6^A modification in AD model. **(A)** 10 μM Aβ inhibited the total m^6^A content in the SH-SY5Y cells (*n* = 3). **(B)** qRT-PCR showed mRNA levels of methyltransferases (METTL3, METTL14, and WTAP) after 10 μM Aβ treatment in the SH-SY5Y cells (*n* = 3). **(C)** qRT-PCR showed unchanged mRNA levels of demethylases (ALKBH5, FTO) after 10 μM Aβ treatment in the SH-SY5Y cells (*n* = 3). **(D)** Western blotting analysis showed declined protein levels of METTL3 after 10 μM Aβ treatment in the SH-SY5Y cells (*n* = 3). Data were expressed as mean ± SEM, and unpaired *t*-test was performed. *p < 0.05.

To elucidate whether the induction of Aβ changes m^6^A modification, the total m^6^A RNA level was analyzed in the SH-SH5Y cells after Aβ treatment. The m^6^A level in total RNA was found to be down-regulated by Aβ (Figure 2A). Because the level of m^6^A modification is mediated by methyltransferases (“m^6^A writers”) and demethylases (“m^6^A erasers”), the expression of “m^6^A writers” and “m^6^A erasers” was detected in cells. Results showed RNA expression of most methyltransferases in both SH-SY5Y cells and rat hippocampal neurons, such as METTL14 and WTAP remained unchanged compared to the control group (Figures 2B,C and Supplementary Figure 1). Meanwhile, the demethylases ALKBH5 and FTO only decreased apparently in rat hippocampal neurons but not in SH-SY5Y cells after Aβ treatment (Figures 2B,C and Supplementary Figure 1). Besides, the expression of METTL3, a core component of “m^6^A writers,” was significantly down-regulated in both SH-SY5Y cells and rat hippocampal neurons after 10 μM Aβ treatment, as shown by real-time quantitative polymerase chain reaction (RT-qPCR) and Western blotting (Figures 2B,D). These findings indicate that METTL3 expression was down-regulated, which decreased the total m^6^A level in Aβ-induced cell models.

### Methyltransferase-Like 3 Up-Regulated Activity-Regulated Cytoskeleton Associated Protein Expression Through N6-Methyladenosine Modification

**FIGURE 3:**
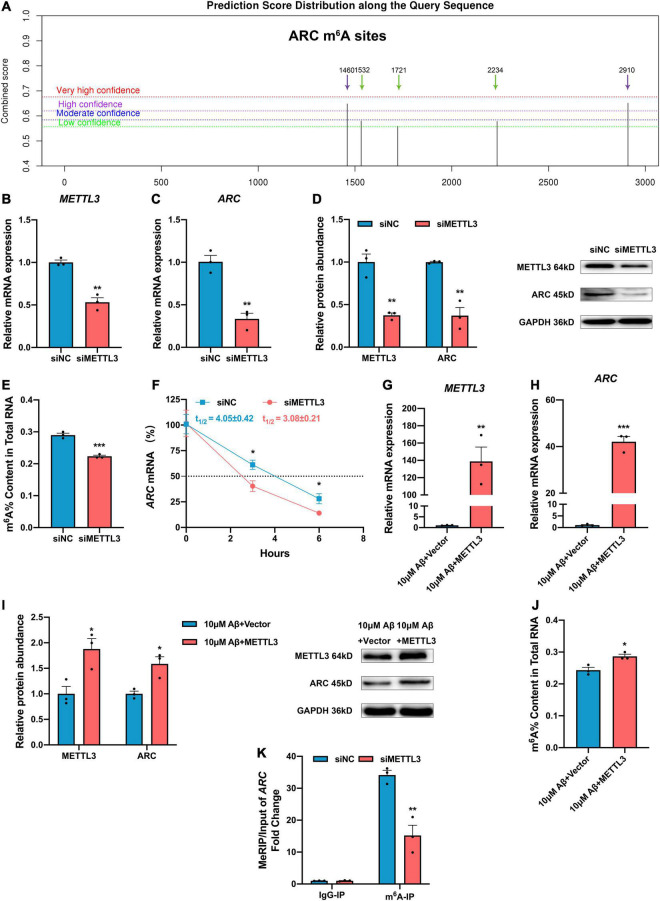
Methyltransferase-like 3 (METTL3) promotes ARC expression and increased *ARC* mRNA stability *via* m^6^A modification. **(A)** Potential sites for m^6^A modification in the sequence of *ARC* gene by SRAMP. **(B)** qRT-PCR showed the successful knockdown of METTL3 (*n* = 3). **(C)** *ARC* mRNA was down-regulated with the knockdown of METTL3 (*n* = 3). **(D)** Western blotting analysis showed decreased protein levels of METTL3 and ARC in the SH-SY5Y cells transfected with METTL3 siRNA (*n* = 3). **(E)** Loss of METTL3 declined the total m^6^A level (*n* = 3). **(F)** RNA stability assay showed the half-life of *ARC* mRNA with METTL3 knockdown (*n* = 3). **(G)** qRT-PCR showed the successful overexpression of METTL3 after treating with 10 μM Aβ (*n* = 3). **(H)** *ARC* mRNA was retrieved by over-expressed METTL3 after 10 μM Aβ treatment (*n* = 3). **(I)** Western blotting analysis showed increased protein levels of METTL3 and ARC in the SH-SY5Y cells transfected with METTL3 cDNA after 10 μM Aβ treatment (*n* = 3). **(J)** Total m^6^A level increased with rescued METTL3. **(K)** MeRIP-qPCR showed reduction of m^6^A modification in *ARC* by siMETTL3 (*n* = 3). Data were expressed as mean ± SEM. **(B–E,G–J)** Unpaired *t*-test, and **(F,K)** two-way ANOVA were performed. *p < 0.05, **p < 0.01, ***p < 0.001.

Activity-regulated cytoskeleton associated protein expression is mainly regulated by epigenetic modification (Penner et al., 2011; Singh and Thakur, 2018; Myrum et al., 2020), but whether m^6^A modification can regulate ARC expression remains unclear. Of note, the *ARC* RNA (NM_015193.5) has five potential m^6^A modification sites according to the Sequence-based RNA Adenosine Methylation Sites Predictor [SRAMP (see text footnote 2), Figure 3A]. To further investigate the effect of METTL3 on ARC expression, METTL3 expression was successfully knocked down with siRNA in the SH-SY5Y cells (Figure 3B). As expected, the mRNA and protein expressions of ARC were both significantly down-regulated after METTL3 knock-down (Figures 3C,D). Additionally, the knockdown of METTL3 suppressed the m^6^A level of total RNA (Figure 3E). To investigate the stability of *ARC* mRNA, the SH-SY5Y cells were treated with transcription inhibitor actinomycin D (ActD), and results showed a significant decline in the stability of *ARC* mRNA after METTL3 knockdown (Figure 3F). Then, METTL3 was overexpressed in the SH-SY5Y cells by cDNA transfection and 10 μM Aβ treatment (Figure 3G). Results showed that METTL3 overexpression retrieved ARC expression (Figures 3H,I), as well as the m^6^A level of total RNA (Figure 3J). To validate that *ARC* mRNA is the target gene of METTL3 for m^6^A modification, MeRIP–qPCR was performed. Results showed that the knockdown of METTL3 dramatically reduced the m^6^A level of *ARC* mRNA enriched by m^6^A specific antibody (Figure 3K). These findings suggest that METTL3 plays an essential role in the up-regulation of ARC expression.

### Activity-Regulated Cytoskeleton Associated Protein Expression Was Up-Regulated in an N6-Methyladenosine-YTH Domain Family, Member 1-Dependent Manner

**FIGURE 4:**
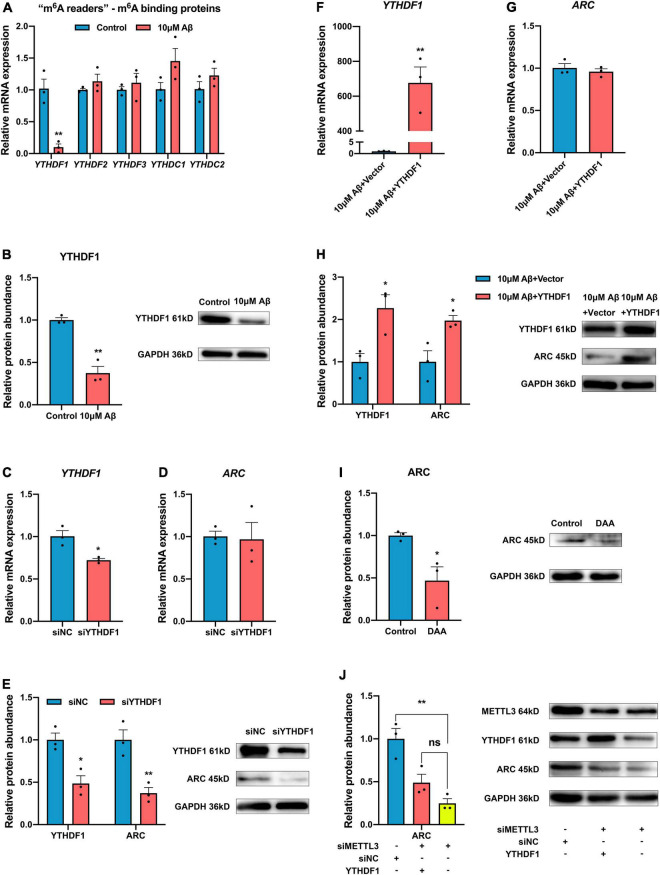
YTH domain family, member 1 (YTHDF1) up-regulates ARC expression through a m^6^A-dependent manner. **(A)** qRT-PCR showed the mRNA levels of m^6^A binding proteins (YTHDF1-3, YTHDC1-2) after 10 μM Aβ treatment in the SH-SY5Y cells (*n* = 3). **(B)** Western blotting analysis showed declined protein levels of YTHDF1 after 10 μM Aβ treatment (*n* = 3). **(C)** qRT-PCR showed successful knockdown of YTHDF1 in the SH-SY5Y cells (*n* = 3). **(D)** *ARC* mRNA showed no variance with the knockdown of YTDHF1 (*n* = 3). **(E)** Western blotting analysis showed decreased protein levels of YTHDF1and ARC in the SH-SY5Y cells transfected with YTHDF1 siRNA (*n* = 3). **(F)** qRT-PCR showed the successful overexpression of YTHDF1 after treatment with 10 μM Aβ (*n* = 3). **(G)** YTHDF1 overexpression did not influence *ARC* mRNA after 10 μM Aβ treatment (*n* = 3). **(H)** Western blotting analysis showed increased protein levels of YTHDF1 and ARC in the SH-SY5Y cells transfected with YTHDF1 cDNA after 10 μM Aβ treatment (*n* = 3). **(I)** m^6^A inhibitor DAA treatment decreased ARC protein expression (*n* = 3). **(J)** Overexpression of YTHDF1 didn’t increase ARC expression without the existence of METTL3 (*n* = 3). Data were expressed as mean ± SEM. **(A–I)** Unpaired *t*-test, and **(J)** one-way ANOVA were performed. ^ns^*p* > 0.05, *p < 0.05, **p < 0.01.

The m^6^A modified RNA needs to be recognized by “m^6^A readers”. The expression of “m^6^A readers” was detected in the present study. Results showed that except the RNA expression of YTHDC1 only decreased in rat hippocampal neurons, other “m^6^A readers” YTHDC2, YTHDF2, and YTHDF3 remained unchanged but YTHDF1 expression decreased obviously after Aβ treatment in both SH-SY5Y cells and rat hippocampal neurons (Figures 4A,B and Supplementary Figure 1). After YTHDF1 knockdown, the *ARC* mRNA remained unchanged (Figures 4C,D), while the protein expression of ARC significantly decreased (Figure 4E). After Aβ treatment, the YTHDF1 overexpression could rescue ARC protein expression but not mRNA expression (Figures 4F–H). In addition, ARC protein expression was further detected after treatment with 3-deazaadenosine (DAA), an inhibitor of S-adenosylhomocysteine hydrolase that could inhibit the total level of m^6^A RNA. Results showed decreased protein expression of ARC (Figure 4I). Moreover, overexpression of YTHDF1 didn’t influence ARC protein expression without the existence of METTL3 (Figure 4J). Our results indicate that m^6^A modification mediated by METTL3 facilitates ARC expression through YTHDF1-dependent mRNA translation.

## Discussion

This study reveals that METTL3 epigenetically activates ARC through a METTL3-m^6^A-YTHDF1-dependent mechanism, which plays a critical role in the m^6^A epitranscriptomic change in Aβ-induced cell models. This knowledge provides new molecular targets for AD therapy.

Although ARC has been shown to be involved in the pathogenesis of AD, the relationship between Aβ and ARC is still unclear. Some studies have reported that ARC expression reduces or is absent in AD models or patients, while other studies indicate that ARC expression increases in the medial prefrontal cortex of AD patients (Penner et al., 2011; Wu et al., 2011). In our study, ARC expression declined in AD patients and Aβ treatment cellular models. Unlike other IEGs, ARC is a downstream effector of numerous signal cascades but is not a transcriptional factor. Additionally, ARC expression is dynamically regulated by neuronal activity variation in the brain and may reflect recent neuronal activity. The expression of ARC was low in the untreated or unstimulated SH-SY5Y cells or primary neurons, and the ARC expression was reported to be primarily induced by electrical stimulations or neuronal activities in cellular or animal models (Wall et al., 2018; Zhang et al., 2018). However, in some researches, the ARC expression was directly examined in the baseline (Myrum et al., 2017; Jang et al., 2019). Therefore, ARC expression may be different under distinct conditions or distinct time points. In the early stage of AD, Aβ accumulation may cause persistent aberrant neuronal activity to induce ARC expression, which then induces Aβ generation (Wu et al., 2011). However, with increasing amounts of amyloid-β precursor protein or Aβ deposition, neuronal hyperexcitation declines neuronal activity apparently (Rice et al., 2019; Zott et al., 2019), which may reduce ARC expression. The dynamic change in ARC expression may account for the discrepancies among available studies. Considering the fact that the ARC expression is influenced by neuronal activities, SH-SY5Y cells could act as an ideal *in vitro* model. And the cell viability assay indirectly represented the cellular metabolic activity. Therefore, we examined the viability of the SH-SY5Y cells, and the results showed decreased viability with the Aβ treatment but didn’t change with the transfection of METTL3/YTHDF1 or the DAA treatment (Supplementary Figure 2). These results indicated that ARC expression was not only influenced by cell viability after Aβ treatment, but also regulated by m^6^A modification, which does not affect cell viability.

Although ARC expression is mainly regulated by transcription and translation, the transcriptional and post-translational modifications, such as SUMOylation, ubiquitination, and phosphorylation, of ARC have been reported previously to drive synaptic plasticity in a stimulus-dependent manner. More importantly, the epigenetic modification of ARC, including DNA methylation, histone methylation, and histone acetylation, can also control its activity and stability to fine-tune synaptic activity (Penner et al., 2011; Singh and Thakur, 2018; Myrum et al., 2020). Recently, m^6^A modification has emerged as a crucial modifier in epigenetics, due to its similar characteristics to epigenetic DNA and histone modification (Zhang et al., 2020). Notably, more than 80% of methylated mRNA bases in the adult brain were modified by m^6^A modification. And Shafik et al. (2021) observed high levels of m^6^A modification in the early and late stages in mouse brain, which attributed to the most profound gene expression changes that occur at these stages. Besides, m^6^A modification could regulate synaptic plasticity, learning and memory, and stress response in ischemic stroke or AD. For example, Chokkalla et al. (2019) found that m^6^A levels increased after transient focal ischemia, which may be mediated by loss of m^6^A demethylase FTO; Shafik et al. (2021) observed down-regulated levels of m^6^A and METTL3; and Zhang et al. (2018) observed expression of ARC decreased in METTL3-knockout mice, reducing memory consolidation ability. However, the precise mechanism of dynamic m^6^A modification of *ARC* is still not clear. Our results showed that the major “m^6^A writer” (METTL3) and “reader” (YTHDF1) modified ARC. The m^6^A modification is a complex process that requires multitudes of enzymes. Notably, the total level of m^6^A modification depends on the dynamic balance between methyltransferase “writers” and demethylase “erasers.” In our study, the expression of METTL3 decreased after Aβ treatment, while other “m^6^A writers” or “m^6^A erasers” showed no variations, which was similar to the results in AD patients (Huang et al., 2020; Zhao et al., 2021). This phenomenon may be attributed to the different expression of m^6^A enzymes under variant conditions; however, the precise mechanism needs to be further investigated. For the “m^6^A writers,” METTL3 and METTL14 form a stable core complex: METTL3 acts as the catalytic subunit, while METTL14 acts as an auxiliary component to facilitate RNA binding. Our results showed that the m^6^A level of total RNA ultimately decreased. This phenomenon indicates that the loss of METTL3 expression is more crucial to the m^6^A modification.

Thus, the modified m^6^A mRNA needs “m^6^A readers” in order to take effect. Different “m^6^A readers” have been reported to be related to various fates of mRNA, including the stability, splicing, and translation of RNA. METTL3-mediated m^6^A methylation promotes ARC expression, and thus, we hypothesize that ARC is a target of YTHDF1. YTHDF1 promotes the translation of m^6^A methylated RNA. Therefore, we focused on the roles of METTL3 and YTHDF1 in *ARC* mRNA stability. Our results showed that YTHDF1 interacted with the m^6^A modified sites on the *ARC* mRNA and controlled the *ARC* mRNA stability *via* an m^6^A-dependent pathway to impact ARC expression. Recently, Ries et al. (2019) reported that YTHDF1 could enhance the phase separation potential of m^6^A modified mRNA, thus further studies could explore whether YTHDF1 mediate the phase separation of ARC.

Lots of studies have emphasized that ARC acts as a key factor for AD due to its role in synaptic plasticity and long-term memory consolidation. In this research, we mainly investigated the mechanism of ARC expression in Aβ treatment cellular models. However, AD is too complicated to be just mimicked by the Aβ treatment, the precise relationship among the Aβ and m^6^A enzymes still needs to be intensively investigated in primary hippocampal neurons or animal models of AD. And it’s our limitation that experiments *in vitro* could explain how ARC affects synapses and cognitive functions after METTL3/YTHDF1 mediated methylation modification. In vivo experiments, such as patch clamp or Morris Water Maze, would be performed to explore the effects of METTL3/YTHDF1 mediated methylation modification on synaptic plasticity and cognitive function in the following study.

**FIGURE 5:**
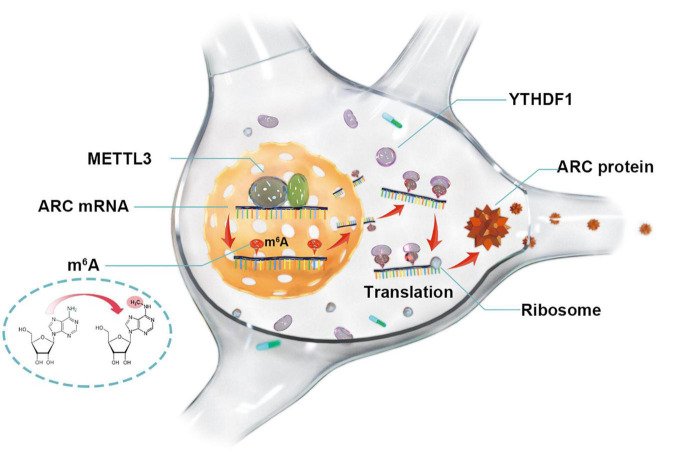
Methyltransferase-like 3 promotes ARC expression *via* a YTHDF1-mediated m^6^A manner.

In summary, our study suggests that METTL3 is essential for ARC expression and provides an m^6^A-dependent regulatory mechanism (Figure 5). The combined network of m^6^A “writer” (METTL3), “reader” (YTHDF1), and “target” (ARC) consists of an innovative m^6^A-dependent gene regulatory pathway in the epigenetics. Retrieving METTL3/YTHDF1 to enhance ARC expression might be considered a new molecular strategy for the treatment of AD due to the important roles of ARC in the formation and consolidation of memory.

## Data Availability Statement

The original contributions presented in the study are included in the article/Supplementary Material, further inquiries can be directed to the corresponding authors.

## Ethics Statement

This study was approved by the ethics committee of Tenth People’s Hospital, Tongji University School of Medicine, Shanghai (No. 16 SHDSYY-2020-0971). No human studies are presented in this manuscript.

## Author Contributions

CX and HH performed the study and wrote the manuscript. MZ and PZ analyzed the data. MF, ZL, and XL designed and funded the experiment. All authors read and approved the final manuscript.

## Conflict of Interest

The authors declare that the research was conducted in the absence of any commercial or financial relationships that could be construed as a potential conflict of interest.

## Publisher’s Note

All claims expressed in this article are solely those of the authors and do not necessarily represent those of their affiliated organizations, or those of the publisher, the editors and the reviewers. Any product that may be evaluated in this article, or claim that may be made by its manufacturer, is not guaranteed or endorsed by the publisher.

## Footnotes

1
http://www.alzdata.org

2
http://www.cuilab.cn/sramp

## Supplementary Material

Supplementary Figure 1 The RNA expression of m6A enzymes in the primary neuron after Aβ treatment. qRT-PCR showed mRNA levels of methyltransferases (METTL3, METTL14, WTAP), demethylases (ALKBH5, FTO), and m6A binding proteins (YTHDF1-3, YTHDC1-2) after 10 μM Aβ treatment in the primary neurons.

Supplementary Figure 2 CCK-8 was performed to detect the cell viability under different conditions. **(A)** 10 μM Aβ inhibited the cell viability in the SH-SY5Y cells significantly. **(B)** Knockdown of METTL3 didn’t influence the cell viability. **(C)** Overexpression of METTL3 after 10 μM Aβ treatment didn’t alter the cell viability. **(D)** Knockdown of YTHDF1 didn’t influence the cell viability. **(E)** Overexpression of METTL3 after 10 μM Aβ treatment didn’t alter the cell viability. **(F)** m6A inhibitor DAA treatment didn’t alter the cell viability.

Supplementary Table 1 The sequences of primers used in the experiments.

Supplementary Table 2 The ARC expression in control and AD patients in the AlzData database. Data was expressed as median [IQR]. IQR = interquartile range. NA = not available. **p* < 0.05, ***p* < 0.01, ****p* < 0.001.

## Abbreviations

DAA: 3-deazaadenosine
AMPA: α-amino -3-hydroxy-5-methyl-4-isoxazolepropionicacid
ActD: actinomycin D
ARC: activity-regulated cytoskeleton-associated protein
ARG3.1: activity-regulated gene 3.1 protein homolog
AD: Alzheimer’s Disease
Aβ: amyloid-beta protein
IEGs: immediate early genes
MeRIP: m^6^A-RNA immunoprecipitation
METTL14: methyltransferase-like 14
METTL3: methyltransferase-like 3
m^6^A: N^6^-methyladenosine
OD: optical density
RT-qPCR: RNA isolation and real-time quantitative polymerase chain reaction
SRAMP: sequence-based RNA adenosine methylation sites predictor
SEM: standard error of the mean
WTAP: WT1 associated protein
YTHDFs: YTH domain family members
YTHDF1: YTH domain family member 1.
