# Supplementary figures and images for: Methyltransferase-Like 3 Rescues the Amyloid-beta protein-Induced Reduction of Activity-Regulated Cytoskeleton Associated Protein Expression via YTHDF1-Dependent N6-Methyladenosine Modification
Source: Front Aging Neurosci. 2022 Apr 25;14:890134. doi: 10.3389/fnagi.2022.890134 (PMC9084913; doi:10.3389/fnagi.2022.890134)

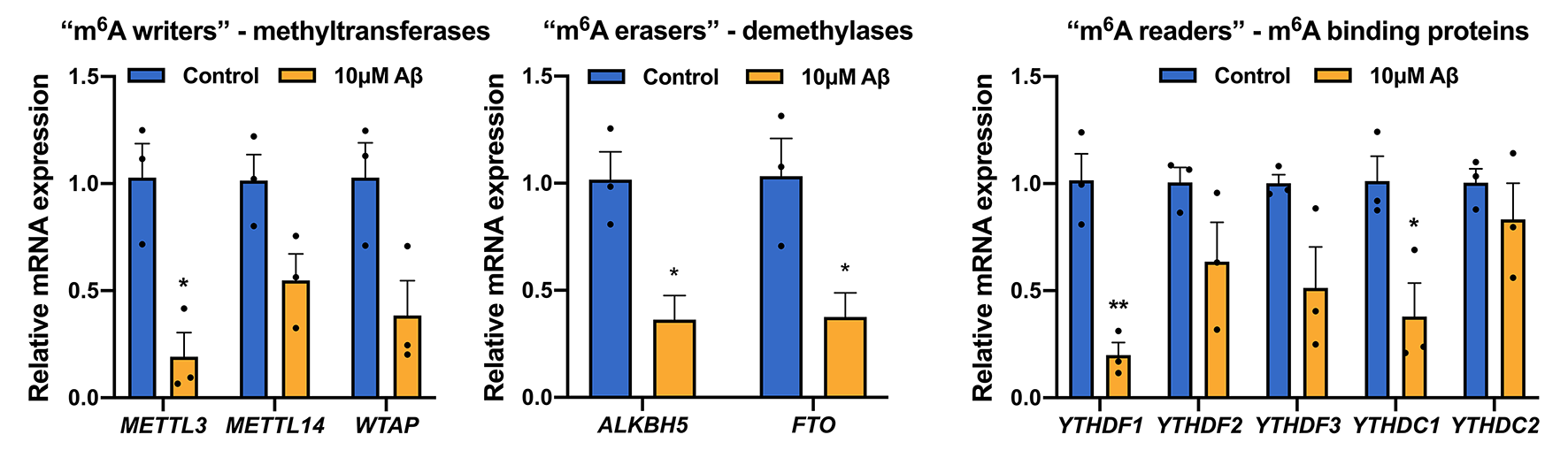

Supplement: Supplementary Figure 1 — The RNA expression of m6A enzymes in the primary neuron after Aβ treatment. qRT-PCR showed mRNA levels of methyltransferases (METTL3, METTL14, WTAP), demethylases (ALKBH5, FTO), and m6A binding proteins (YTHDF1-3, YTHDC1-2) after 10 μM Aβ treatment in the primary neurons. [file Image_1.TIF]

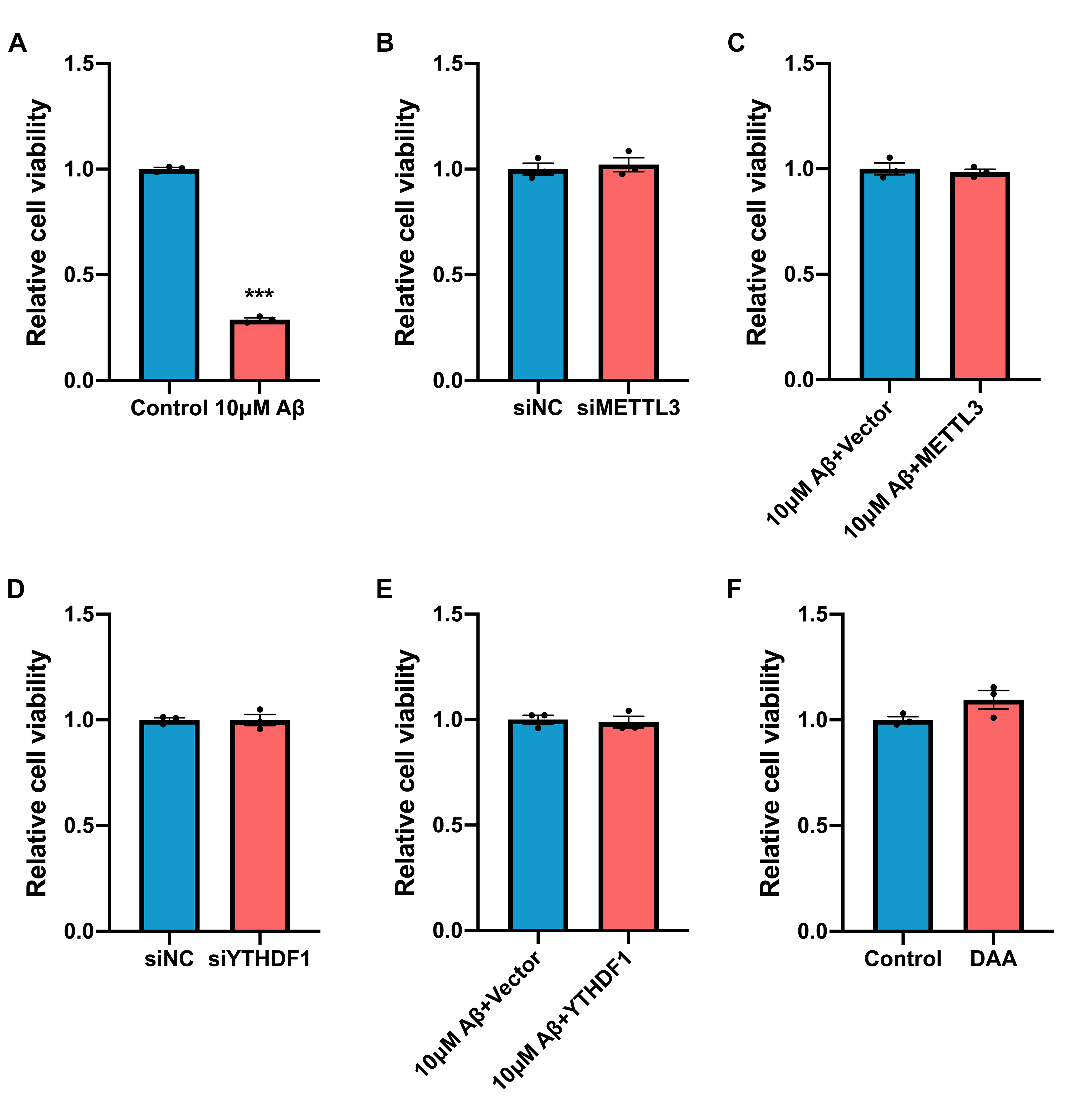

Supplement: Supplementary Figure 2 — CCK-8 was performed to detect the cell viability under different conditions. (A) 10 μM Aβ inhibited the cell viability in the SH-SY5Y cells significantly. (B) Knockdown of METTL3 didn’t influence the cell viability. (C) Overexpression of METTL3 after 10 μM Aβ treatment didn’t alter the cell viability. (D) Knockdown of YTHDF1 didn’t influence the cell viability. (E) Overexpression of METTL3 after 10 μM Aβ treatment didn’t alter the cell viability. (F) m6A inhibitor DAA treatment didn’t alter the cell viability. [file Image_2.PNG]
